# The Action of Medicines in the System, Etc.

**Published:** 1853-12

**Authors:** 


### EDITORIAL.

*The Action of Medicines in the System; or, "On the mode in which Therapeutic agents introduced into the Stomach produce their peculiar effects on the Animal Economy;"*—being the prize Essay, to which the Medical Society of London awarded the Fothergillian gold medal for, *MDCCCLII.* By Frederick William Headland, B. A., M. R. C. S., &c. Philadelphia: Lindsay & Blakiston. 1853. 8 vo. pp. 560.

Among the various departments of medical research, there is no one perhaps of more practical importance, or presenting greater claims to the attention of the Medical Profession, and more earnestly demanding a diligent, careful, and thorough investigation, than the one to which the above Essay is devoted. It is a subject full of interest and importance, not only as contributing in its results to the advancement of Medical Science, but also in its great practical application in the treatment of disease. And its investigations, if conducted upon scientific principles, and with a spirit of patient, but determined inquiry, cannot but be productive of great good. It is a humiliating fact, that this subject, a knowledge of which is so important and essential to the judicious application of therapeutic agents, should have received so little attention from medical men, for while other sciences and other inquiries into the various departments of medical science, have advanced rapidly and with certainty, this has made comparatively but slow and uncertain progress. But few indeed, have given it that earnest and careful study, so essential in the investigation of science. True, classifications of medicines have been made, based upon their observed effects, and various opinions and theories been offered from time to time, in explanation of the modus operandi of medicinal agents. But theories, however ingenious and plausible they may be, without some degree of proof, are comparatively valueless. Facts and facts only should be admitted as constituting the *basis* of our reasoning. Hypotheses may often give to our thoughts and investigations the right direction, and conduct us ultimately to the glittering ore of truth, but too often they are founded in error, and based on false supposition. 'This confounding of assertion with fact,—the mistake of hypothesis for truth' is an error into which many have fallen, and the conclusions deducible from such, must necessarily be incorrect.

Dr. Headland in the dissertation before us seems to be fully sensible of this great error. He says: "in such illogical and incorrect reasoning is to be found the true source of a multitude of errors."

"Every branch of study," *Whately's Elements of Logic*, as quoted by our author, "which can at all claim the character of a science requires two things: 1st. A correct ascertainment of the data from which we are to reason; and 2d. Correctness in the process of deducing conclusions from them."

That the subject is one of magnitude, and presenting many difficulties, all will admit;—the 'difficulty, as Dr. Headland truly says, 'depending mainly on the variety and complexity of the proof required to establish any one point with absolute certainty." Probably on no question in the wide range of medical inquiry, have scientific men differed more widely, than on the *modus operandi* of medicines. This diversity of opinion, is evidence in itself, of the difficulty of furnishing proof sufficient to establish the correctness of what may seem plausible and reasonable in theory.

But, notwithstanding all these difficulties and uncertainties, some progress has been made, some error exposed, and truth elicited. It is most earnestly to be hoped, that the period has passed, when men are seemingly content with simply knowing the ultimate effect of a remedy. While the causes and symptoms of disease are being daily unfolded to our view and better understood, let those who feel a further interest in the advancement of medical science, and the alleviation of human suffering, emulate the undertaking of Dr. Headland, and ere long, "instead of our present indecision and uncertainty on many points, we shall find ourselves eminently qualified to wage the conflict with disease, being skilled in that science whose name bespeaks its importance,—the Science of ***Therapeutics.***

Dr. Headland in the 1st chapter of his book, after briefly speaking of the extent and difficulties attending his undertaking, proceeds to an exposition of the plan pursued, dividing his subject into *ten distinct propositions*, forming the basis of his essay, each of which he will attempt to prove separately.

As the principal conclusions to which he arrives are embodied in these, we present them.

PROP. I. That the great majority of medicines must obtain entry into the blood, or internal fluids of the body, before their action can be manifested.

PROP. II. That the great majority of medicines are capable of solution in the gastric or intestinal secretions, and pass without material change, by a process of absorption, through the coats of the stomach and intestines, to enter the capillaries of the Portal system of veins.

PROP. III. That those medicines which are completely insoluble in water, and in the gastric and intestinal juices, cannot gain entrance into the circulation.

PROP. IV. That some few remedial agents act locally on the mucous surface, either before absorption, or without being absorbed at all. That they are chiefly as follow :—

*a.* Irritant Emetics.
*b.* Stomach Anæsthetics.
*c.* Irritant Cathartics.

PROP. V. That the medicine, when in the blood, must permeate the mass of the circulation, so far as may be required to reach the parts on which it tends to act.

That there are two possible exceptions to this rule :—

*a.* The production of sensation or pain at a distant point.
*b.* The production of muscular contraction at a distant point.

PROP. VI. That while in the blood the medicine may undergo changes, which in some cases may, in others may not, affect its influence. That these changes may be—

*a.* Of Combination.
*b.* Of Reconstruction.
*c.* Of Decomposition.

PROP. VII. That a first class of medicines, called Hæmatics, act while in the blood, which they influence. That their action is permanent.

1. That of these some, called Restoratives, act by supplying, or causing to be supplied, a material wanting; and may remain in the blood.
2. That others, called Catalytics, act so as to counteract a morbid material or process; and must pass out of the body.

PROP. VIII. That a second class of medicines, called Neurotics, act by passing from the blood to the nerves or nerve-centres, which they influence. That they are transitory in action.

1. That of these some, called stimulants, act so as to exalt nervous force, in general or in particular.
2. That others, called Narcotics, act so as first to exalt nervous force, and then to depress it; and have also a special influence on the intellectual part of the brain.
3. That others again, called Sedatives, act so as to depress nervous force, in general or in particular.

PROP. IX. That a third class of medicines, called Astringents, act by passing from the blood to muscular fibre, which they excite to contraction

PROP. X. That a fourth class of medicines, called Eliminatives, act by passing out the blood through the glands, which they excite to the performance of their functions.

It will be observed that the *first* four of these propositions, relate to the conduct of medicines after their introduction into the stomach and before their passage into the blood; that the fifth is simply an extenuation of the first, while in the sixth, it is asserted, that medicines undergo certain changes in the system.

The concluding and most important of all, treat of the *action* of medicines in the system, or 'of the various modes in which they may operate in the *cure* of disease.'

From these it will be seen that Dr. H. divides medicines into our great groups or classes.

In relation to the 1st class,—*Hæmatics*,—our author observes that they are the most important of all medicines, their action being particularly directed towards the blood itself. Medical authors, with few exceptions, have been very backward to acknowledge the existence of medicines of this description. And yet they have been obliged to confess, that there is a set of remedies which they call "Alteratives", whose action is slow and more durable than any others. "But, whatever their subsequent action, they exert a primary and apparant influence on the blood itself. A little reflection will convince us that these remedies are more efficient than any others that can be selected out of the armory of physic."

Of this class of medicines Dr. H. makes two divisions,—viz:

*Restoratives and Catalytics.* The former act by supplying to the blood a material wanting, as Iron in Anæmia. This division is again divided into six orders,—1st. Alimenta; 2d. Acida; 3d. Alkalia; 4th. Tonica; 5th. Chalybeata; 6th. Solventia; each of which is considered separately, and the important medicinal agents embraced by each, enumerated.

In relation to Tonics, of which Quina, may be taken as the type of the whole order, Dr. H. considers that they are not in the first instance Neurotic medicines as some are disposed to contend. He says, "The action of other nerve-medicines is distinguished by the following signs. It is quick, and very rapidly follows the administration of a substance. It is transient and does not endure. It requires no particular state, but takes place in health: thus Alcohol stimulates and intoxicates both healthy and sickly, and Digitalis would subdue a Hercules. Most Neurotics are capable of acting without entry into the blood at large; mere contact with the nerves, as when they are applied externally, being sufficient for their action on those nerves" * * * Now is the primary action of Tonics distinguished by the above signs? Are they quick and sudden in action? Is their effect transitory? Is it evidenced in health as well as in disease? Do they act on the superficial nerves, when applied to them? In each case the answer must be a negative." As additional evidence to prove that Tonics are blood medicines, and should be classed as Restoratives. Dr. H. proceeds next to show, that those disorders in which their use is attended with the greatest benefit, are *blood-diseases.* There are few we imagine, who will give the subject a moment's careful reflection, but will be convinced that this is true. In the condition of simple anæmia, we observe a deficiency in the hematosin of the blood, an element natural and essential to the healthy body, indicating the use of chalybeate medicines. Simple Debility, arising from whatever cause, is traceable to a want of some material, essential to the healthy condition of this fluid. It follows protracted disease, miasmatic influences, and whatever contributes to impoverish the blood, which while they arrest or interfere with the assimilative and building up processes, tend to destroy and remove those elements upon which the strength and tonicity of the system depends. There is a loss of *vital power*, and the consequent existence of a loose and lax condition of muscular fibre. To restore the one and give tone and firmness to the other, requires an improvement in the quality and condition of the blood. This is the office of Tonics, their influence being most marked. But stimulants in such cases are not only of no benefit, but of positive injury. They never increase or exalt *vital* force only when its deficiency or want is occasoned by a loss of *nervous* energy, as exhibited in depression arising from some sudden or accidental cause. We cannot be too careful in distinguishing between *vital force* and *nerve force.* Too often we fear, they are regarded and treated as identical. And the same remark is equally applicable to Tonics and Stimulants. Hence the frequent administration of the latter, when the former alone are indicated, and vice versa. There may be a difficiency or loss of *vital* power as observed in simple Debility, arising from waste and excretion of important elements in the blood, by which it has become impoverished, while *nerve* force may remain normal. Here the use of Tonics is indicated ; but where it follows sudden depression or loss of *nerve* force, and upon which it depends, they are of little or no avail. Here Stimulants are demanded to arouse nerve energy which is weighing down the vitalforces.

Our author's views on the peculiar action of Quina are new and interesting, but our limited space willn otadmit of their notice here. To the Second Division of Hæmatic medicines Dr.H. applies the name *Catalytica*, on the supposition that their action in the blood results in destroying or counteracting certain morbid agencies as mercury in syphilis. This division is divided into eight orders, named according to the several morbid states in which they are used, viz. : 1st. Antiphlogistica ; 2d. Antisyphilitica ; 3d. Antiscrobulosa ; 4th. Antiarthritica ; 5th. Antiscorbutica ; 6th. Antiperiodica; 7th. Anticonvulsiva; 8th. Antisquamosa, each of which is considered separately, and the more important remedies, whose peculiar action and influences are expressed in the above orders, are enumerated.

From the want of space in the present number of the Journal, we are compelled, unexpectedly, to omit any further notice of this Division, and also of three remaining classes of medicine, Neurotics, Astringents and Eliminatives.

The fourth chapter, "*on some of the more important medicines in particular*" must likewise be omitted. But we may have occasion hereafter to speak of these, and of some views there advanced. We have peruse Dr. Headland's book with pleasure and profit, and we most cheerfully commend it to our professional brethren, believing that in their perusal of it they will be both instructed and benefited. Occasionally our author has indulged in loose and indefinite expressions, and we think erroneous assertions, but nevertheless, we welcome it as an undertaking of the right kind,—a step in the right direction, and we hope and trust that the facts it presents and suggestions made, will awaken a deeper interest and a stronger desire to know more of the *modus operandi* of medicine. P.

